# Knowledge and beliefs about antibiotics among people in Yogyakarta City Indonesia: a cross sectional population-based survey

**DOI:** 10.1186/2047-2994-1-38

**Published:** 2012-11-23

**Authors:** Aris Widayati, Sri Suryawati, Charlotte de Crespigny, Janet E Hiller

**Affiliations:** 1 Faculty of Pharmacy, Sanata Dharma University Yogyakarta Indonesia, Kampus III, Paingan Maguwoharjo, Depok, Sleman Yogyakarta, Indonesia; 2 Faculty of Medicine, Gadjah Mada University, Yogyakarta, Indonesia; 3 School of Nursing, University of Adelaide, Adelaide, Australia; 4 School of Population Health, University of Adelaide, Adelaide, Australia; 5 Faculty of Health Sciences, Australian Catholic University, Melbourne, Australia

**Keywords:** Knowledge, Beliefs, Antibiotics, Self medication

## Abstract

**Background:**

Misconceptions about antibiotic use among community members potentially lead to inappropriate use of antibiotics in the community. This population-based study was aimed at examining common knowledge and beliefs about antibiotic use of people in an urban area of Indonesia.

**Methods:**

The population of the study was adults (over 18 years old) in Yogyakarta City. A cluster random sampling technique was applied (N = 640). Data were collected using a pre-tested questionnaire and analyzed using descriptive statistics and correlation.

**Results:**

A total of 625 respondents was approached and 559 respondents completed the questionnaire (90% response rate). Out of 559 respondents, 283 (51%) are familiar with antibiotics. Out of 283 respondents who are familiar with antibiotics, more than half have appropriate knowledge regarding antibiotic resistance (85%), allergic reactions (70%), and their effectiveness for bacterial infections (76%). Half these respondents know that antibiotics ought not to be used immediately for fever (50%). More than half have incorrect knowledge regarding antibiotics for viral infections (71%). More than half believe that antibiotics can prevent illnesses from becoming worse (74%). Fewer than half believe that antibiotics have no side effects (24%), that antibiotics can cure any disease (40%), and that antibiotic powders poured onto the skin can quickly cure injuries (37%). Those who are uncertain with these beliefs ranged from 25% to 40%. Generally, these respondents have moderate knowledge; where the median is 3 with a range of 0 to 5 (out of a potential maximum of 5). Median of scores of beliefs is 13 (4 to 19; potential range: 4 to 20). The results of correlation analysis show that those with appropriate knowledge regarding antibiotics would also quite likely have more appropriate beliefs regarding antibiotics. The correlation is highest for those who are male, young participants, with higher education levels, and have a higher income level.

**Conclusions:**

Misconceptions regarding antibiotic use exist among people in this study. Therefore, improving appropriate knowledge regarding antibiotic use is required.

## Background

People’s misconceptions of antibiotics can potentially lead to inappropriate self medication with either prescribed or non-prescribed antibiotics [1]. A review about antibiotic use in developing countries by Radyowijati and Haak [2] reported that people believed antibiotics as “an extraordinary medicine” or “a powerful medicine” or “a strong medicine” which are able to prevent and cure any diseases or symptoms. Misconceptions and lack of basic knowledge about antibiotic use have also been reported by several studies across populations in both developed and developing countries [3-12]. Patients’ demand for antibiotic prescription and the practice of using antibiotics without prescription by community members is influenced by such misconceptions [6,8,13].

Knowledge and beliefs are social cognitive factors at an individual level that influences health-related behavior, including the behavior of using antibiotics. Knowledge by itself is not enough to change behavior, but does play an important role in shaping beliefs and attitude regarding a particular behavior [14]. Consequently, in the context of antibiotic use, inappropriate knowledge of using antibiotics correctly potentially leads to misconceptions regarding such use. Given that inappropriate use of antibiotics in the community continues to be a significant problem in both developed and developing countries [5,15]; reducing misconceptions regarding antibiotic use among the community members is imperative.

Information on knowledge and beliefs regarding antibiotic use in the developed countries in particular among general people has been widely presented [3,6,7,16]. However, similar knowledge relating to developing world settings, including in Indonesia, is scarce [15]. Therefore, this present study is aimed at describing knowledge and beliefs about antibiotic use among people in an urban area of Indonesia.

## Methods

This study was part of a study assessing self medication with antibiotics in Yogyakarta City Indonesia. Some details regarding methods, in particular sample selection, pre-testing the questionnaire, and data collection, have been presented elsewhere [17]. The study area was Yogyakarta City, Indonesia with a population density of about 14,000 persons per square kilometres in 2010. Yogyakarta City is known as a multiethnic city in Indonesia with the Javanese descent predominates [18]. Ethics approval was provided by the Human Research Ethics Committees at The University of Adelaide Australia (H-145-2009; RM No: 9508) and research permit was issued by the Government of Yogyakarta City Indonesia (Pemerintah Kota Yogyakarta Dinas Perizinan: 070/1970/5328/34).

This cross-sectional population-based survey involved respondents over 18 years who were randomly selected using a cluster random sampling method. The sample size adjusted for cluster design was 640. The sampling process involved the sub-districts in Yogyakarta City which were randomly selected. Households were randomly selected from every sub-district chosen. One family member was randomly selected from every household chosen.

The questionnaire was presented in Bahasa Indonesia, (i.e. the official language of the Republic of Indonesia). There was one dichotomized question to assess participants’ familiarity regarding antibiotics, i.e. “Are you familiar with antibiotics? (Yes/No)”. Then, the “Yes” response was verified, i.e. “If yes, please mention the name/s of the antibiotic/s”. Questions regarding knowledge were assessed using “Yes”, “No”, and “Don’t know” responses. A five-point Likert Scale, (i.e. strongly disagree – disagree - neither disagree nor agree – agree – strongly agree) was used for responses to questions on beliefs. Items in the questionnaire were structured based on published articles where people’s knowledge and beliefs about antibiotics in various countries were assessed [19-23]. The validity of the items in the questionnaire was assessed by a group of local experts. The clarity of language and the user-friendly structures of the questionnaire were pre-tested with five people who have similar characteristics with the study population. The tests resulted in some minor revisions. Details of the questions regarding knowledge (five items) and beliefs (four items) applied in this study are provided in Table 1. There were also questions about demographic and socio-economic characteristics of respondents.

**Table 1:** Items of knowledge and beliefs about antibiotic use self administered to respondents of the survey of self medication with antibiotics in Yogyakarta City Indonesia

**Questions of knowledge and beliefs**	
Knowledge:	
K1:	Antibiotics must be taken as soon as we have fever
K2:	Antibiotics can treat viral infections
K3:	Antibiotics can treat bacterial infections
K4:	People can be allergic to antibiotics
K5:	When antibiotics are taken for the wrong indication this leads to antibiotic resistance
Beliefs:	
B1:	I believe that antibiotics can cure any diseases
B2:	I believe that antibiotics can prevent any illnesses from becoming worse
B3:	I believe that an injury to the skin can be cured quickly by pouring antibiotic powders onto the injury
B4:	I believe that antibiotics do not have any side-effects

The pre-tested questionnaire was self-administered to the respondents who consented to participate in this study during March to May 2010. The respondents were assured that their participation was voluntary and anonymous.

The data were digitally stored and analyzed using SPSS (Statistical Package for the Social Sciences) version 17. Only data of the participants who were able to correctly mention the name of one or more antibiotics was included in the data analysis (the inclusion criteria of data). Those who were not familiar with antibiotics were not required to answer the questions about knowledge and beliefs about antibiotics. These cases were coded as missing values and were dropped from the analysis. To assess potential bias due to these missing values, characteristics of respondents of the excluded data were compared with the included data using Chi-Square test.

The correct responses of the knowledge items were “No” for K1 and K2 and “Yes” for K3 to K5. A total of the correct responses were calculated to show the scores of overall knowledge (ranged from 1 to 5). The overall performance of knowledge was stated as poor (below the median), moderate (at the median), and adequate (above the median) [24].

Regarding the belief items, scores of 1, 2, 3, 4, and 5 were assigned respectively to each option - strongly disagree, disagree, neither agree nor disagree, agree, and strongly agree. Agreeing to the belief items (B1 to B4) was considered as inappropriate beliefs. The overall grade of the beliefs was approached using the total scores of those four belief items (ranged from 4 to 20). As per overall knowledge categorizations, the overall beliefs were stated as appropriate if the scores were below the median line, as moderate if they were at the line, and as inappropriate if they were above the line [24].

Data about demographic and socio-economic characteristics of respondents are reported as a percentage and median. The characteristics include gender, age, education achievement, and family income levels. Correlation analysis was conducted to examine the relationships between knowledge and beliefs. The strength of the correlation coefficients for two different groups (i.e. by gender - male and female; by age - younger: below the median line of age and older: above the median line; by education - lower: senior high school or less and higher: college or university degree; and by income - lower: less than US $ 150 and higher: US $ 150 or more) were also compared. The strength of the correlations was stated as weak at rho = 0.10 to 0.29; moderate at rho = 0.30 to 0.49; and strong at rho = 0.50 to 1.0 using confidence level of 95% (p < 0.05) [25].

## Results

A total of 559 respondents returned the completed questionnaires (90% response rate). Out of 559 respondents, 283 (51%) are familiar with antibiotics and are able to mention the name of antibiotics correctly. Those who are not familiar with antibiotics are 276 respondents. Data from these 283 respondents were analysed.

The socio-economic and demographic characteristics of the respondents are presented in Table 2. Most participants are male (62%). The median of age is 41 years (range: 18 to 88). The majority had completed the senior high school (41%) and is in the lowest income level (i.e. less than US $ 150/month) (45%). Across the socio-demographic characteristics the proportions of those who were excluded (missing data) are not significantly different from those who were included, i.e. gender, X^2^ (1, n = 559) =0.24, p = 0.62, phi = 0.02; age, X^2^ (1, n = 559) = 0.24, p = 0.62, phi = 0.02; and education level, X^2^ (1, n = 559) =3.1, p = 0.08, phi = 0.09.

**Table 2:** Demographic and socio-economic characteristics of respondents of self medication with antibiotics survey in Yogyakarta City Indonesia

**Demographic and socio-economic characteristics**	**Total respondents; N: 559**	
	**Number (percentage) of respondents who are familiar with antibiotics**	**Number (percentage) of respondents who are not familiar with antibiotics**
	**n: 283**	**n: 276**
Gender:		
Female	108 (38)	141(51)
Male	175 (62)	134 (49)
Did not mention	-	1 (0.3)
Age in years:		
Less than 24	24 (8)	32 (11)
24 to 54	209 (74)	149 (54)
54 to 64	33 (12)	49 (18)
More than 64	17 (6)	46 (17)
Median (range)	41 (18 to 88)	43 (18 to 80)
Marital status:		
Married	200 (71)	167 (61)
Unmarried/single	58 (21)	65 (23)
Widow/widower	24 (8)	41 (15)
Did not mention	-	4 (1)
Household’s income per month:		
Less than US $ 150	127 (45)	138 (50)
US $ 150 to US $ 300	91 (32)	86 (31)
US $ 300 to US $ 800	29 (10)	24 (9)
More than US $ 800	9 (3)	2 (1)
Did not mention	27 (10)	26 (9)
Highest education achievement:		
University	90 (32)	71 (26)
Senior high school	115 (41)	92 (33)
Junior high school	33 (12)	37 (14)
Elementary school	21 (7)	28 (10)
Did not mention	24 (8)	48 (17)
Current employment/status:		
Unemployed	94 (33)	69 (25)
Employed	134 (47)	124 (45)
Did not mention	55 (20)	83 (30)
Medical insurance holder:		
Yes	146 (51)	116 (42)
No	129 (46)	149 (54)
Did not mention	8 (3)	11(4)

Note: US $ 1 ~ Rp. 8.200 (Indonesian currency in May 2010); unemployed, i.e.: housewife, retirement, student, jobless; employed, i.e.: private employee, civil servant, entrepreneur, labor.

**Figure 1:**
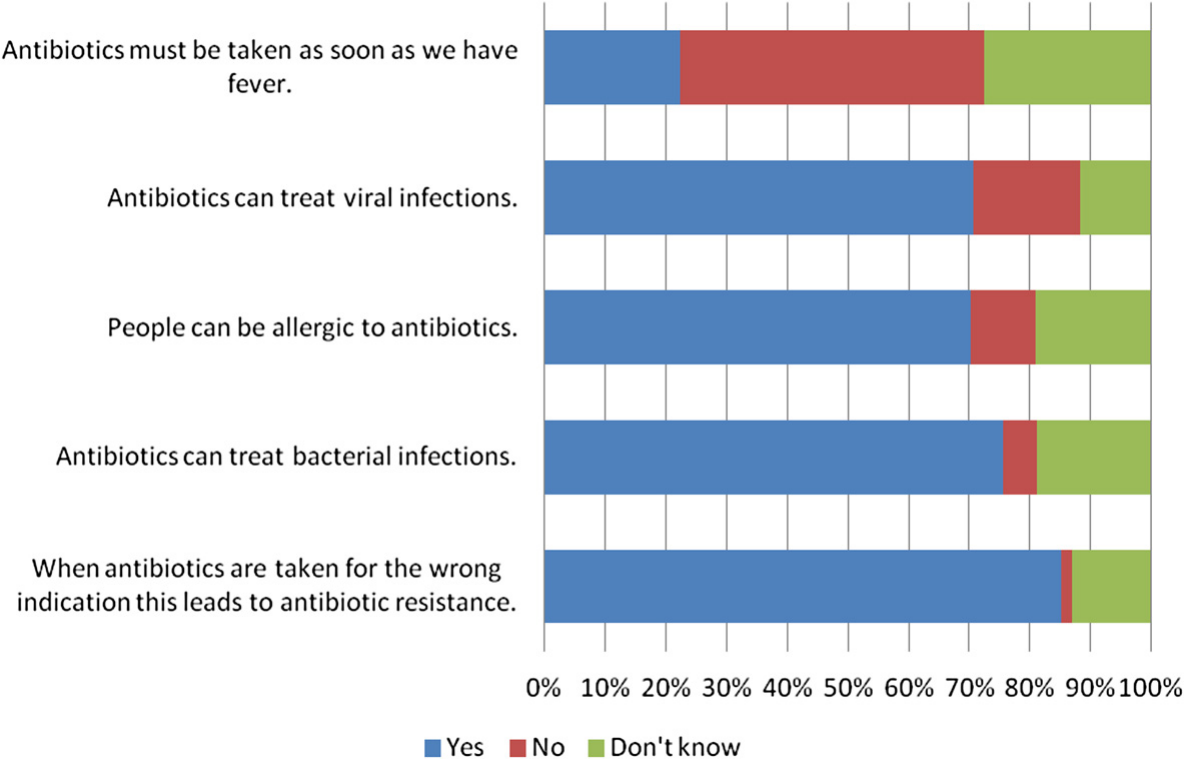
Knowledge about antibiotic use among people in Yogyakarta City Indonesia.

As described in Figure 1, most people in this study (85%) are aware that indiscriminate use of antibiotics leads to antibiotic resistance. Further, most of the participants are able to correctly answer that bacterial infections can be treated by antibiotics (76%), that people can be allergic to antibiotics (70%), and that antibiotics must not be used as soon as they have fever (50%). On the other hand, most of participants (71%) have incorrect knowledge regarding the use of antibiotics for viral infections. The median of overall scores of knowledge is 3; ranged from 0 to 5 (a potential maximum of 5). Regarding level of knowledge, 31% of respondents are at the poor level of overall scores of knowledge, 35% have moderate level of knowledge, and 34% have adequate knowledge.

**Figure 2:**
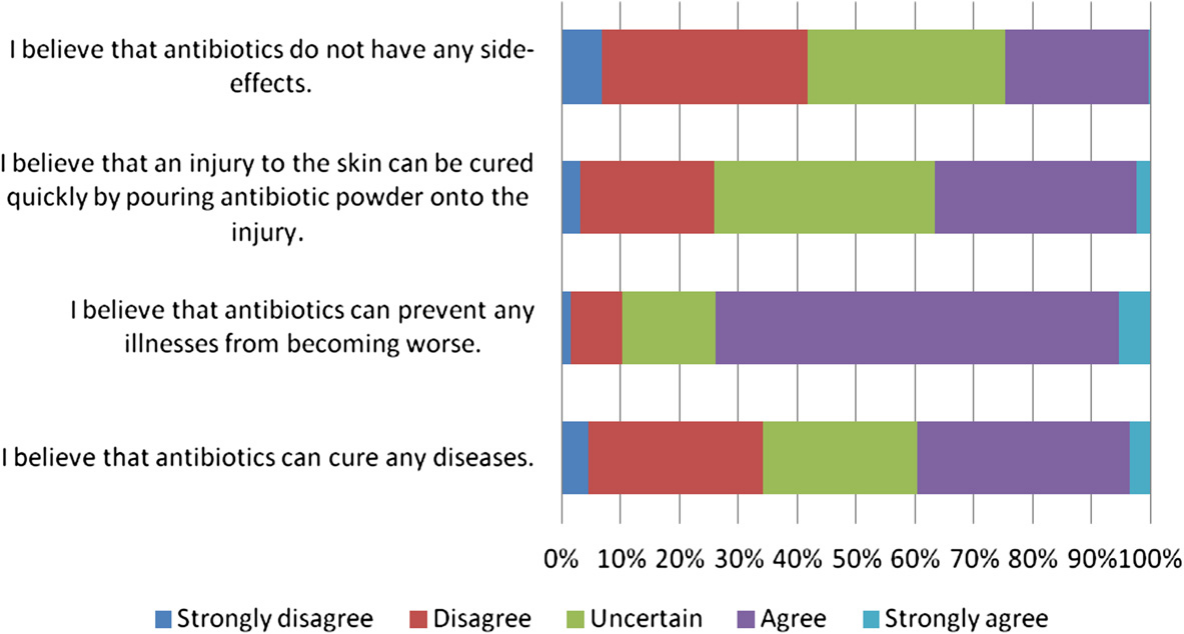
Beliefs about antibiotic use among people in Yogyakarta City Indonesia.

As described in Figure 2, most participants (74%) believe that antibiotics can prevent any diseases from becoming worse. On the other hand, fewer than half believe that antibiotics have no side effects (24%), that antibiotics can cure any illnesses (40%), and that antibiotics can cure skin injuries quickly when they are poured onto the wounds (37%). However, those who neither agree nor disagree with these beliefs ranged from 25% to 40%. Median of the overall scores of beliefs is 13; ranged from 4 to 19 (a potential range of 4 to 20). Percentage of participants with appropriate belief is 29%; moderate belief is 46%; inappropriate belief is 25%.

Association between knowledge and beliefs is moderate and negative, rho = −0.261, n = 283, p < 0.01; in which high scores of knowledge associated with lower scores of beliefs; meaning that the more appropriate knowledge they have, the less misconceptions they have.

As seen in Table 3, the correlations between knowledge and belief for male is higher than female (r = −0.328 and −0.214); for younger is higher than older (r = −0.323 and −0.212); for those with the higher education achievement is higher than those with the lower education levels (r = −0.2345 and −0.197); and for those with the higher income levels is higher than those with the lower income (r = −0.317 and r = −0.145).

**Table 3:** Comparing the correlation coefficients of knowledge and beliefs about antibiotic use by gender, age, education achievements, and economic levels among people in Yogyakarta City Indonesia

**Socio-demographic variables**	**Correlation coefficients of knowledge and beliefs**
Gender	
Male	−0.328**
Female	−0.214
Age	
Younger	−0.323**
Older	−0.212
Education	
Lower	−0.197
Higher	−0.235**
Income	
Lower	−0.145
Higher	−0.317**

Notes: Spearman’s rho correlation; N = 283.

**. Correlation is significant at the 0.01 level (2-tailed).

## Discussion

Overall results of this study show that most participants had moderate to adequate knowledge regarding antibiotic use. They were aware with the risks of antibiotic use; for example, regarding antimicrobial resistance, allergic and possible side effects. Most of them knew that antibiotics are effective for bacterial infections, but had inappropriate knowledge regarding antibiotics’ effectiveness for viral infections. In terms of beliefs about antibiotic use, overall they expressed beliefs that antibiotics can prevent any symptoms/diseases from becoming worse. Only a few believed that antibiotics have no side effects; that antibiotics can cure any diseases; and that antibiotics can be used effectively to cure skin injuries by pouring the powders onto the wounds. Furthermore, the relationships between knowledge and beliefs suggest that the more appropriate their knowledge about the use of antibiotics; the fewer misconceptions they will have regarding the effectiveness of antibiotics.

There are some limitations in this study, in particular regarding the methods used. Firstly, given that the study involved the population of an urban area of Indonesia, results of this study would apply more to urban people who are mostly literate, are able to easily get access to the mass media, and possibly have received more information about antibiotics than those in rural areas. Secondly, a recognized source of error in studies of antibiotic use involving lay people is whether the participants are able to differentiate antibiotics from other types of medicines [2]. However, participants who were not familiar with antibiotics were excluded from this study, to minimize bias. Given that nearly half of the cases were excluded, checking was done to assess bias due to these missing values. As mentioned earlier there are no significant differences in any of the socio-demographic characteristics between those who were included and those who were excluded.

Generally, lay people in both developed and developing countries are aware that antibiotics are effective for bacterial infections [6,11,26]. Interestingly, there is inconsistency in the literature regarding the appropriate knowledge among the community members about the effectiveness of antibiotics in treating viral infections [3,6,26,27]. Furthermore, inconsistent information also exists in terms of people’s knowledge about other therapeutic effects of antibiotics; for example the immediate use of antibiotics for treating a fever or treating skin injuries by pouring antibiotics powders onto them [6,11,28]. Evidence mentioned earlier demonstrates that such misconceptions regarding therapeutic effects of antibiotics do exist among the general public. These facts give evidence to confirm that people are not able to differentiate the types of causal agents of infectious disease, (e.g.: bacteria, viruses, fungal) and they have very limited knowledge regarding the basic mechanism of how the antibiotics work.

In contrast to those misconceptions, people in this study had a sufficient knowledge regarding the risks of using antibiotics, such as antibiotic resistance and allergies. These findings are in line with most other studies from elsewhere [6,11,27-31], but in contrast to what was reported by the European study [5]. People in this study were familiar with the term “resistance” although information on resistance is not usually provided when purchasing antibiotics [32]. However, it should be noted that when lay people talk about “resistance”, this term could mean human resists to antibiotics rather than microorganisms to antibiotics. Another possibility is that they might perceive the term of resistance as “something dangerous”. Although people may have inappropriate understanding regarding the meaning of resistance, these findings, somehow, indicate their awareness regarding the risks of antibiotic use.

In this present study, association between beliefs and knowledge is negative and moderate. This means that the more appropriate knowledge people have, the less misconceptions they have. This correlation is likely to be held by the population and is higher for male, for younger, for those with higher education, and for those with higher income level. This finding suggests that women, older people, and those with lower formal education and income levels could be prioritized in any efforts for reducing misconceptions about antibiotic use.

Inappropriate use of antibiotics exists among Indonesians, as is indicated by the previous studies [17,23]. Appropriate beliefs regarding antibiotic use may lead people becoming more aware of the disadvantages of using antibiotics inappropriately. Based on the findings of this present study, further studies are suggested. Firstly, a similar study needs to be conducted in rural areas of Indonesia, as this present study represents the urban people. Secondly, a better understanding is needed on the extent to which beliefs can influence people using antibiotics in inappropriate ways; for example using antibiotics without medical consultation. Thirdly, it is imperative to develop a sustainable intervention program to reduce misconceptions of antibiotic use and to increase public’s awareness about the risks of inappropriate use of antibiotics.

## Conclusions

We believe that this study is useful in describing people’s knowledge and beliefs regarding antibiotic use among Indonesians in urban areas. The findings may be useful to help develop intervention to decrease misconceptions regarding antibiotic use and to increase people’s awareness regarding the risks of inappropriate use of antibiotics in the community.

## Competing interests

The authors declare that they have no competing interests.

## Authors’ contributions

All authors contributed to conception and design of the study. AW carried out data collection, data analysis, data interpretation and drafted the manuscript. JEH, CdeC, and SS checked and clarified data analysis and data interpretation; and revised draft of the manuscript critically. All authors read and approved the final manuscript.
